# Improved Production of Induced Pluripotent Stem Cells Using Dot Pattern Culture Plates

**DOI:** 10.1089/ten.tec.2023.0068

**Published:** 2023-09-14

**Authors:** Yoshiki Nakashima, Hiroki Iguchi, Eiko Shimizu, Minh N.T. Le, Kenta Takakura, Yuta Nakamura, Teruhiko Yanagisawa, Rutvi Sanghavi, Satoshi Haneda, Masayoshi Tsukahara

**Affiliations:** ^1^ Research and Development Center, Kyoto University Center for iPS Cell Research and Application Foundation (CiRA Foundation), Kyoto, Japan.; ^2^ Life Science Development Center Advanced Technology Institute R&D Center Corporate, SEKISUI CHEMICAL CO., LTD., Osaka, Japan.; ^3^ Kyoto University iCeMS Institute for Integrated Cell-Material Sciences, Kyoto, Japan.

**Keywords:** induced pluripotent stem cells, culture system, dot pattern culture plates

## Abstract

The rate of cell proliferation is a crucial factor in cell production under good manufacturing practice (GMP) control. In this study, we identified a culture system for induced pluripotent cells (iPSCs) that supports cell proliferation and viability and maintains the cells in an undifferentiated state even at 8 days after seeding. This system involves the use of dot pattern culture plates that have been coated with a chemically defined scaffold which has high biocompatibility. Under cell starvation conditions, where medium exchange was not performed for 7 days or where the amount of medium exchange was reduced to half or a quarter, iPSC viability and lack of differentiation were maintained. The rate of cell viability in this culture system was greater than generally obtained by standard culture methods. The cells in this compartmentalized culture system could be induced to differentiate in a controlled and consistent manner: differentiation of endoderm occurred in a controlled and consistent manner: endoderm, mesoderm, and ectoderm could be consistently induced to differentiate in the cultures. In conclusion, we have developed a culture system that supports high viability in iPSCs and allows their controlled differentiation. This system has the potential for use in GMP-based production of iPSCs for clinical purposes.

**Impact statement:**

Currently, the production of induced pluripotent cells (iPSCs) for clinical use is manually performed in a clean room according to a protocol developed in academia. However, the standard protocol for iPSCs requires passage within 6 days. In the present study, we constricted the area in which cells attached on cell culture plates. We found that this change enabled iPSCs to be passaged at intervals of 7 days or longer. Such a change would greatly benefit the scheduling of commercial cell production.

## Introduction

To support the development of cell-based drugs derived from human induced pluripotent stem cells (hiPSCs),^1,2^ we are currently striving to improve the processes necessary for industrialized production of clinical iPSCs.^3^ Colony morphology of iPSCs is widely recognized as an important indicator of the cell quality; in academic laboratories, round colonies with a diameter of about 1 mm are generally used for passage, whereas, for clinical iPSCs, colonies with a diameter of about 0.5 mm are generally passaged.

The production schedule for clinical iPSCs and the cell quality obtained is significantly affected by the time (days) from seeding to passage of colonies. Five other factors are also of considerable importance: (1) cell seeding density^4^ (cells/mL); (2) colony diameter^5^ (/mm); (3) frequency of medium change (times/day) or (mL/cm^3^/h); (4) dissolved oxygen concentration^6^ (mg/L); (5) number of days between passages (passages/X days). These factors are affected by the iPSC type (A), cell proliferation rate (B), medium composition^7^ (C), scaffold material types^8^ (D), and scaffold material shape (E). Factors (1) to (5) are culture conditions that can be set by a manufacturing process manager. However, factors (A) to (E) also markedly influence the clinical iPSC production schedule and quality of cells produced.

In the present study, to manage the combination of the above variables and factors as data, certain culture conditions with consideration of the production of clinical iPSCs were set, after which “(2) colony diameter” was standardized. As the base culture material, pattern plates (Sekisui Chemical Co., Ltd., Osaka, Japan) with four dot sizes (μm) coated with chemicals were used. After cell seeding, the rates of differentiated cells, apoptotic cells, necrotic cells, and live cells on each plate were assessed. The present study also compared the outcome of cultures on a fibronectin motif with cultures using a standard scaffold, iMatrix-511. The use of a fibronectin motif^9^ as a coating material was tested for the first time.

The present study investigated the optimum colony diameter to develop a method for culturing iPSCs with an artificially defined colony diameter. Standardizing the colony diameter and number of iPSCs in the culture vessel will facilitate iPSC culture utilizing data processing based on numerical prediction and contribute to culture automation.

## Materials and Methods

### Reagents

StemFit AK03N was obtained from Ajinomoto Healthy Supply Co., Inc. (Tokyo, Japan). iMatrix-511 was obtained from Matrixome, Inc., (Osaka, Japan). Dulbecco's modified Eagle's medium (DMEM), DMEM/nutrient mixture F-12 Ham 1:1 (DMEM-F12), XAV939 a Wnt pathway inhibitor, 45% (w/v) D(+)-glucose solution, and 200 mM L-glutamine solution ( × 100) were purchased from FUJIFILM Wako Pure Chemical Corporation (Osaka, Japan). The glycogen synthase kinase-3 inhibitor CHIR 99021-CT 99021 was obtained from Axon Medchem LLC (Reston,VA). A 10 mM solution of the Rho associated (ROCK) inhibitor Y-27632, D-PBS(-), and 0.5 M-EDTA solution (pH 8.0) were obtained from Nacalai Tesque (Kyoto, Japan). LDN193189 dihydrochloride, an inhibitor of the bone morphogenetic pathway (BMP), was obtained from Tocris Bioscience (Bristol, United Kingdom).

RPMI 1640 Medium, B-27™ Supplement, minus insulin, B-27 Supplement (50 × ), TrypLE™ Select Enzyme (1 × ), GlutaMAX™ Supplement, Neurobasal™ Medium, N-2 Supplement (100 × ), and Insulin-Transferrin-Selenium-Sodium Pyruvate (ITS-A) (100 × ) were obtained from Thermo Fisher Scientific K.K. (Kanagawa, Japan). 100 × Non-Essential Amino Acids (NEAAs) were obtained from MP Biomedicals, LLC (Santa Ana, CA). Recombinant Human Activin A was obtained from BioLegend, Inc., (San Diego, CA). Recombinant Human BMP-4 was obtained from PeproTech (Cranbury, NJ). SB431542, an inhibitor of the activin receptor-like kinase receptors ALK5, ALK4, and ALK7, was obtained from Cayman Chemical Company (Ann Arbor, MI). Kyoto Probe-1 (KP-1), a fluorescent probe that identifies undifferentiated iPSCs, was obtained from Goryo Chemical, Inc., (Sapporo, Japan).

### Preparation of dot pattern cell culture plates

Chemically defined scaffold (CDS) and CDSD plates were provided by Sekisui Chemical Co., Ltd. Six-well Tissue Culture (TC)-treated plates (Corning Incorporated, Corning, NY) were coated with a synthetic polymer scaffold containing a fibronectin motif. Dot patterns were printed with a synthetic polymer ink diluted in ethanol. The coating process was performed using an inkjet dispenser. After completing the drying and sterilizing process, CDS and CDSD plates were prepared. Regarding the coating of iMatrix-511 on each well/six wells, with the following reagents added per well: 9.6 μL of iMatrix-511 (0.5 μg/μL) +1.5 mL PBS.

### Maintenance culture of hiPSCs

The hiPSC line 15M66 was established by Shinya Yamanaka (CiRA Foundation) and obtained from CiRA Foundation (Kyoto, Japan). A standard protocol was used to culture iPSCs (CiRA_Ff-iPSC_protocol_Eng_v140310; https://www.cira.kyoto-u.ac.jp/j/research/img/protocol/Ff-iPSC-culture_protocol_E_v140311.pdf).

### Cell differentiation assays

#### Cardiomyocyte differentiation

(1) hiPSCs that had been cultured on laminin-coated (iMatrix-511) were added to six-well plates (5 × 10^4^ cells/well); StemFit AK03N with 10 μM Y27632 was added to each well to obtain a final volume of 1.5 mL in each well. The cells were incubated at 37°C and 5% CO_2_. (2) On days −6, −4, and −2 before differentiation, the medium was replaced with 1.5 mL of room-temperature StemFit AK03N per well. (3) On day 0 of differentiation, 12 μM CHIR99021 in 4 mL of RPMI/B-27 without insulin was added to each well. (4) After 24 h (day 1 of differentiation), the medium in each well was replaced with 4 mL of room-temperature RPMI/B-27 without insulin. The plates were returned to the incubator. (5) On day 3 of differentiation 2 μM XAV939 was added to each well. (6) On day 5 of differentiation, the medium was removed from each well and 4 mL room-temperature RPMI/B-27 without insulin was added to each well. The plate was then returned to the incubator. (7) On day 7 of differentiation, and every 3 days thereafter, the medium in each well was replaced with 4 mL fresh RPMI/B-27 and then incubated.

#### Endoderm differentiation

The protocol for induction of endoderm differentiation has previously been described.^10^

#### Neuroprogenitor cell differentiation

(1) hiPSCs (5 × 10^4^ cells/well) were added to laminin-coated (iMatrix-511) six-well plates in StemFit AK03N with 10 μM Y27632 to obtain a final volume of 1.5 mL in each well. The plates were then placed in an incubator at 37°C, 5%CO_2_. (2) At days −6, −4, and −2 before induction of differentiation, the medium was removed and replaced with 1.5 mL of room-temperature StemFit AK03N per well. (3) Neural induction medium (NIM) was prepared by combining equal amounts of neurobasal medium and DMEM/F12; the medium was supplemented 0.3% glucose, 2 mM L-Glutamine, 1 × N-2, 0.5 × B27, and 1 × ITS-A. On day 0 of differentiation, the medium in each well was removed and replaced with 4 mL NIM supplemented with SB431542 (10 μM) and LDN193189 (100 nM). The plates were replaced into the incubator. (4) The hiPSC colonies were allowed to differentiate to neuroprogenitor cells for 7 days, with the medium being replaced every other day.

### Cell proliferation assays

Cell proliferation was measured using a Countess cell counter (Thermo Fisher Scientific K.K.).

### Cell death assay

The rate of death of hiPSCs was quantified using an Apoptotic/Necrotic/Healthy Cell Detection Kit (PromoCell, Heidelberg, Germany) according to the manufacturer's instructions. Images were recorded using a BZ-9000 fluorescence microscope (KEYENCE CORPORATION). The ImageJ software program was used to quantify the area of the light emitted in the image.

### Real time polymerase chain reaction and use of a quantitative polymerase chain reaction array

RNA was prepared using an RNeasy Mini Kit (QIAGEN N.V., Hilden, Germany) and LunaScript^™^ RT SuperMix Kit (New England Biolabs, Inc., Ipswich, MA) according to the manufacturers' instructions. Real-time polymerase chain reaction was performed using a StepOnePlus system (Life Technologies, Carlsbad, CA). For the mRNA expression analysis, a GeneQuery^™^ Human quantitative polymerase chain reaction (qPCR) Array (HUMAN Pluripotent Stem Cell Biology: GQH-hPSC, HUMAN Cell Cycle: GQH-CCY, HUMAN Fibroblast Growth Factor Signaling Pathway:GQH-FGF; ScienCell, Carlsbad, CA) was used. The Luna Universal qPCR Master Mix (New England Biolabs, Inc.) was used according to the manufacturer's instructions. The primers used for PCR were as follows:

*undifferentiated ES cells*,
human OCT3/4 (NM_002701.4) 144 bp,
(forward) GACAGGGGGAGGGGAGGAGCTAGG,
(reverse) CTTCCCTCCAACCAGTTGCCCCAAAC;
human *NANOG* (NM_024865.2) 391 bp,
(forward) CAGCCCCGATTCTTCCACCAGTCCC,
(reverse) CGGAAGATTCCCAGTCGGGTTCACC;
human SOX2 (NM_003106.2) 151 bp,
(forward) GGGAAATGGGAGGGGTGCAAAAGAGG,
(reverse) TTGCGTGAGTGTGGATGGGATTGGTG;
*ectoderm*,
human PAX6 (NM_001604.4) 317 bp,
(forward) ACCCATTATCCAGATGTGTTTGCCCGAG,
(reverse) ATGGTGAAGCTGGGCATAGGCGGCAG;
human MAP2 (NM_001039538.1) 212 bp,
(forward) CAGGTGGCGGACGTGTGAAAATTGAGAGTG,
(reverse) CACGCTGGATCTGCCTGGGGACTGTG;
human SOX1 (NM_005986.3) 158 bp,
(forward) ACTCTCTCTGAGGTTCTTTGACTGA,
(reverse) AGCTTTTCATAGTCTGTGCCTCTAA;
*endoderm*,
human SOX17 (NM_022454.3) 608 bp,
(forward) CGCTTTCATGGTGTGGGCTAAGGACG,
(reverse) TAGTTGGGGTGGTCCTGCATGTGCTG;
human FOXA2 (NM_153675.2) 216 bp,
(forward) TGGGAGCGGTGAAGATGGAAGGGCAC,
(reverse) TCATGCCAGCGCCCACGTACGACGAC;
human HNF4A (NM_000457.4) 239 bp,
(forward) GAACAGGAGCTCTTAACTACAGTGG,
(reverse) CTGTCAAGAGTCATGAATTCTCCTT;
*mesoderm*,
human T (NM_001270484.1) 211 bp,
(forward) GCTGAACTCCTTGCATAAGTATGAG,
(reverse) CATCTCTTTGTGATCACTTCTTTCC;
human NKX2.5 (NM_001166175.1) 221 bp,
(forward) GAAATTTTAAGTCACCGTCTGTCTC,
(reverse) AGTAATGGTAAGGGATCCTCGTG;
human TNNT2 (NM_001001432.1) 238 bp,
(forward) ATGAGCGGGAGAAGGAGCGGCAGAAC,
(reverse) TCAATGGCCAGCACCTTCCTCCTCTC;
*housekeeping gene*,
human β-ACTIN (NM_001101.5) 223 bp,
(forward) TGACATTAAGGAGAAGCTGTGCTAC,
(reverse) CTTCATGATGGAGTTGAAGGTAGTT.

### qPCR array

RNA was prepared using a SuperPREP II Cell Lysis & RT Kit for qPCR (Toyobo Co., Ltd., Osaka, Japan) according to the manufacturer's instructions. Real-time PCR was performed using a StepOnePlus system (Life Technologies). For the mRNA expression analysis, a TaqMan Array 96-Well FAST Plate (Human Stem Cell Pluripotency or Human DNA Repair Mechanism; Applied Biosystems) was used with TaqMan™ Fast Advanced Master Mix (Thermo Fisher Scientific, K.K.) according to the manufacturer's instructions. The PCR protocol was as follows: (1) denature at 95°C for 20 s, continue denaturing the double-stranded DNA; (2) anneal primers at 60°C for 20 s, and repeat steps (1)–(2) 40 times.

For the analysis of real-time PCR data using a TaqMan Array 96-Well FAST Plate, 18S, GAPDH, HPRT1, and GUSB were used as housekeeping genes. The maximum CT value was set at 40. The ΔCT for undetected was calculated by subtracting the average of the CT values of the four housekeeping genes from the maximum CT value (40). The ΔCT value of the target was calculated by subtracting the average CT values of the four housekeeping genes from the CT values of the various genes under each culture condition. To calculate the ΔΔCT values of the target, the average ΔCT values of the various genes under control culture conditions were subtracted from the ΔCT values of the various genes under each culture condition. ΔΔCT values were then calculated using the Excel software program (Microsoft Corporation, Redmond, WA).

### Cell staining

Recently, Kyoto probe 1 (KP-1), a fluorescent immunostaining method for staining iPSC-like cells or tissue-specific progenitor cells^11^ that can be generated by the iPSC generation method, was reported.^12^ Staining of live cells was performed using KP-1 (Goryo Chemical, Inc., Hokkaido, Japan). Images were recorded using a BZ-9000 fluorescence microscope (KEYENCE CORPORATION). The ImageJ software program was used to quantify the area of the light emitted in the image.

### Statistical analyses

Statistical analyses were performed using Student's *t*-test to compare two sample means. Comparisons between multiple groups (more than two groups) were performed using a one-way analysis of variance with the StatPlus software program (AnalystSoft, Walnut, CA). Statistical significance was set at *p < 0.05 or **p < 0.01 for all tests. The data shown are representative examples of two independent experiments.

## Results

### Problem of nonuniform colony size in clinical iPSC production

**FIG. 1.**
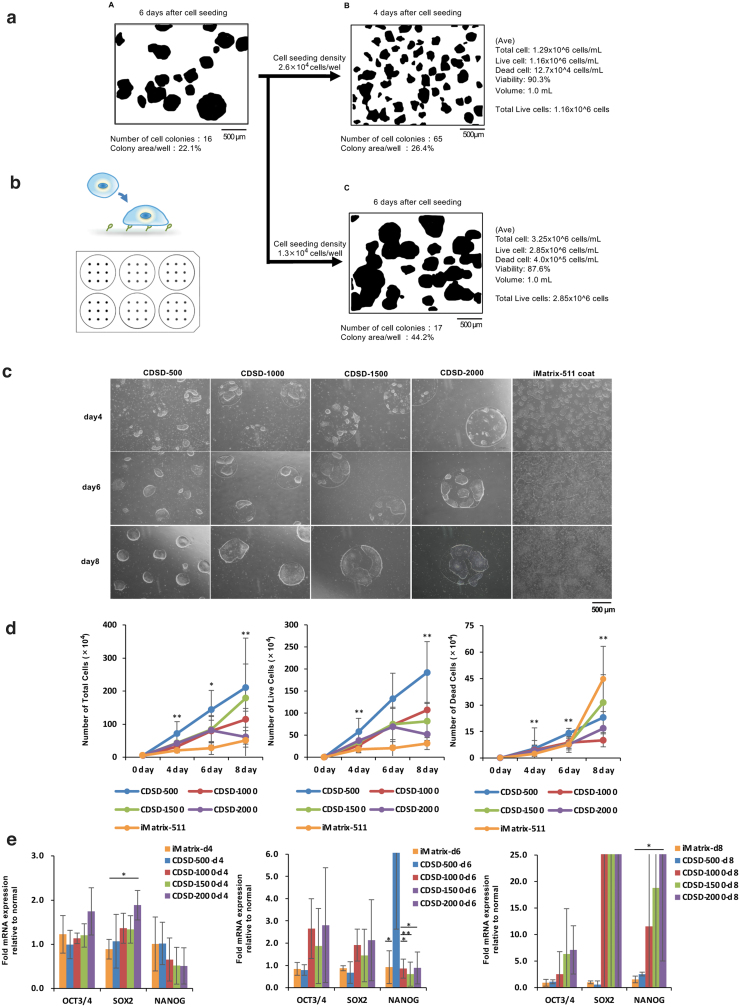
Measurement of culture behavior and cell proliferation behavior on a *dot* pattern plate. **(a)** Clinical iPSCs were seeded at two cell concentrations in a laboratory-based experiment. The figure shows the area of cell proliferation before passage (*left* figure) and the colony growth area on days 4 (*upper right* figure) and 6 (*lower right* figure) after cell seeding. The cell count information following cell detachment is shown on the *right* side of the figure. **(b)** Schematic illustration of a culture plate coated with a synthetic polymer scaffold containing a fibronectin motif. **(c)** Light microscope images of iPSCs (15M66) cultured on CDSD-500, CDSD-1000, CDSD-1500, CDSD-2000, and iMatrix-511-coated plates for 4, 6, and 8 days. Scale bar = 500 μm. **(d)** iPSCs (15M66) cultured on CDSD-500, CDSD-1000, CDSD-1500, CDSD-2000, and iMatrix-511-coated plates for 4, 6, and 8 days. The total number of cells is shown in the *left panel*; the number of live cells is shown in the *middle panel*; the number of dead cells is shown in the *right panel*. Six plates were assessed for each culture condition. Data represent the mean ± SD. *p < 0.05. **p < 0.01. *n* = 6. **(e)** iPSCs (15M66) cultured on CDSD-500, CDSD-1000, CDSD-1500, CDSD-2000, and iMatrix-511-coated plates for 4 (*left panel*), 6 (*middle panel*), and 8 (*right panel*) days. Real-time PCR of human embryonic stem cell markers. Three plates were assessed for each culture condition. *n* = 2. iPSC, induced pluripotent cell.

Currently, the hierarchy of “cell count data > manufacturing schedule management > cell quality control” is emphasized. As cell count data cannot be obtained before cell detachment, then another metric is necessary for controlling the manufacturing process and automating the process management. We focused on standardizing the colony diameter for this purpose. First, we used a cell seeding density of 1.3 × 10^4^ cells/well (six-well plate) to subculture iPSCs on day 6 (Fig. 1a-A, C). However, in the first cell seeding after thawing frozen cells, cell death is likely to occur, so cells are seeded at higher concentrations than ultimately desired. In addition, cells may be seeded at a concentration of 2.6 × 10^4^ cells/well (six-well plate) under conditions where the cell growth rate is not stable. However, if the cell seeding density is high, the number of colonies will increase, so cells may be used for the next passage 4 or 5 days after initial cell seeding (Fig. 1a-B).

As such, the fact that the interval between passages cannot be determined without observing the cells is a heavy burden on manufacturing schedule management. Clinical iPSCs produced in our cell processing facility are seeded into wells (six wells) at a density of 1.3 × 10^4^ cells/well (six-well plate). Therefore, in Figure 1, the cell density was 1.3 × 10^4^ cells/well (six-well plate). However, in this experiment with dot patterns, the seeded cell density was set at ≥5 × 10^4^ cells/well (six-well plate) to ensure that the seeded cells adhered uniformly to the dot patterns. This technology requires the cells to be seeded at a higher density. At a usual cell seeding density, it would probably be difficult for the cells to adhere to the dots in the wells.

Six-well plates chemically coated with dots of a fibronectin motif were custom-made. CDSD-500 plates have a dot size of 500 μm, a total of 638 dots, and a total adhesive area of 1.2 cm^2^. CDSD-1000 plates have a dot size of 1000 μm, a total of 154 dots, and a total adhesive area of 1.2 cm^2^. CDSD-1500 plates have a dot size of 1500 μm, a total of 69 dots, and a total adhesive area of 1.2 cm^2^. CDSD-2000 plates have a dot size of 2000 μm, a total of 40 dots, and a total adhesive area of 1.2 cm^2^ (Fig. 1b).

The experiments performed here were conducted using 15M66 cells, a research cell line for clinical iPSCs. The cells were seeded into CDSD-500, CDSD-1000, CDSD-1500, and CDSD-2000 plates, and optical micrographs were obtained on days 4, 6, and 8. As a control, cells were seeded onto an iMatrix-511-coated plate; images of these cells are presented in the right panels of Figure 1c. On all of the plates, multiple colonies were fused to form dot-sized colonies. On Day 8, a confluent state was present in the dots (Fig. 1c).

Cell count data were obtained on days 4, 6, and 8 after seeding of the cells; these data included the total cell number (Fig. 1d, left figure), number of live cells (Fig. 1d, middle figure), and number of dead cells (Fig. 1d, right figure). Data on cell numbers for the culture using an iMatrix-511-coated plate were obtained based on a six-well plate cell culture area of 9.5 cm^2^ using the standard methods. Since the data in Figure 1 for the experimental plates are based on a cell adhesion area of 1.2 cm^2^, we also show the corrected number for 9.5 cm^2^ for comparison to the control cultures. In all of the dot pattern plates, cell numbers increased to 1 × 10^6^ cells/well after cell seeding, peaking on day 8. Thus, the number of adherent iPSCs in the confluent state for the total adhesion surface (1.2 cm^2^) of the pattern plate was 1 × 10^6^ cells. The number of adherent cells on day 8 of culture was less than 20 times the number of seeded cells. On day 8 of culture, the proportion of dead cells was significantly lower in the dot pattern plates than the iMatrix-511 coated plate (Fig. 1d, right figure).

An mRNA analysis of cells on dot pattern plates was performed on days 4, 6, and 8 and compared to cells seeded on the iMatrix-511-coated plate (Fig. 1e). On day 4, the mRNA levels of the marker genes *OCT3/4* and *SOX2*, which identify undifferentiated cells, were increased on CDSD-2000 cultures compared to the control; the increase was significant for *SOX2*. On day 6, the levels of *OCT3/4* and *SOX2* mRNAs were higher on CDSD-1000, CDSD-1500, and CDSD-2000 plates compared to the control, but the increases were not significant. On CDSD-500 plates, the expression of *NANOG*, a marker of undifferentiated cells, was significantly higher than in the other culture conditions, although there was considerable variation among samples. On day 8, *OCT3/4* and *SOX2* expression was nonsignificantly increased on CDSD-1000, CDSD-1500, and CDSD-2000 plates compared to controls. On CDSD-2000 plates, cells showed significantly higher levels of *NANOG* compared to the control (Fig. 1e, right figure).

### Cell numbers and expression of undifferentiated markers in 7-day cultures without medium exchange

**FIG. 2.**
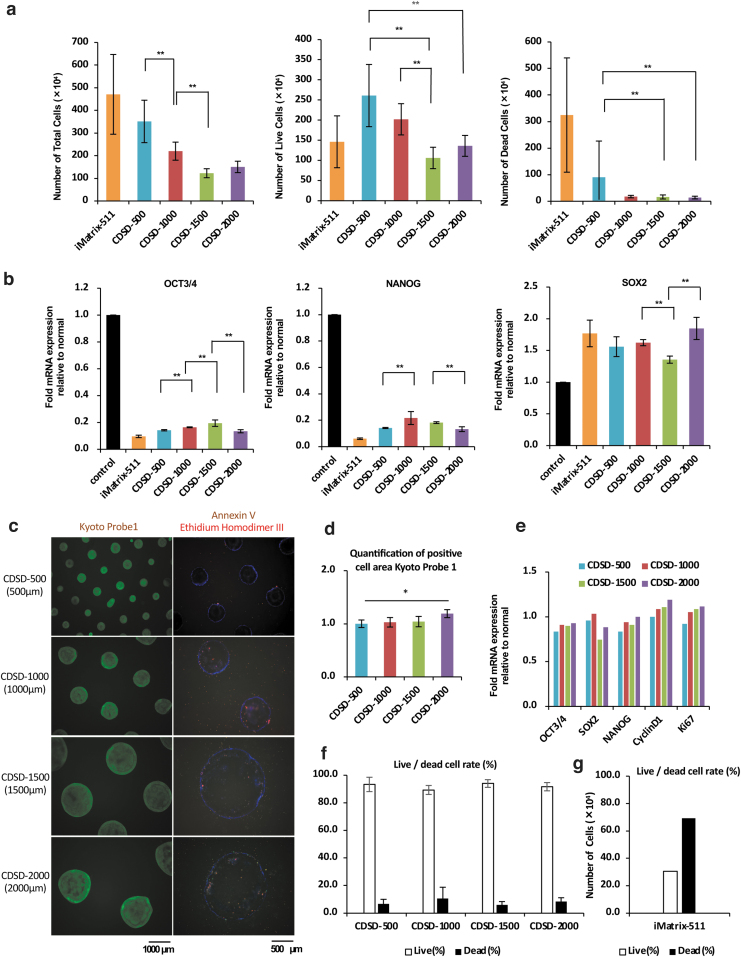
Analysis of cells cultured for 7 days. **(a)** iPSCs (15M66) after culturing on CDSD-500, CDSD-1000, CDSD-1500, CDSD-2000, or iMatrix-511-coated plates for 7 days without medium exchange. The total number of cells (*left panel*), the number of live cells (*middle panel*), and the number of dead cells (*right panel*) are shown. Six plates were analyzed for each culture condition. Data represent the mean ± standard deviation (SD). *p < 0.05, **p < 0.01. *n* = 6. **(b)** iPSCs (15M66) were cultured on CDSD-500, CDSD-1000, CDSD-1500, CDSD-2000, or iMatrix-511-coated plates for 7 days without medium exchange. iPSCs (15M66) seeded on iMatrix-511-coated plates (9.2 μL/well [six wells]) had their medium changed as usual for 7 days, and the mRNA expression on day 7 was used as the control value. They were then subjected to real-time PCR for *OCT3/4* (*left panel*), *NANOG* (*middle panel*), and *SOX2* (*right panel*). Six plates were analyzed for each culture condition. Data represent the mean ± standard deviation (SD). *p < 0.05, **p < 0.01. *n* = 6. **(c)** iPSCs (15M66) were cultured on CDSD-500, CDSD-1000, CDSD-1500, and CDSD-2000 plates for 7 days with medium exchange and then subjected to immunofluorescence staining. *Left panels*: immunofluorescent staining of KP-1, a pluripotency marker. Scale bar = 1000 μm. *Right panels*: immunofluorescent staining for Annexin V, an apoptosis marker, and Ethidium Homodimer III, a marker for dead cells. Scale bar = 500 μm. **(d)** iPSCs (15M66) were cultured on CDSD-500, CDSD-1000, CDSD-1500, and CDSD-2000 plates for 7 days with medium exchange and then subjected to immunofluorescent staining for KP-1, a pluripotency marker. Luminous areas per well were measured. The relative values are indicated. Three plates were analyzed for each culture condition. Data represent the mean ± SD. *p < 0.05. *n* = 3. **(e)** iPSCs (15M66) were cultured on CDSD-500, CDSD-1000, CDSD-1500, and CDSD-2000 plates for 7 days with medium exchange and then used for a real-time PCR analysis of human embryonic stem cell markers and cell cycle markers. One plate was analyzed for each culture condition. *n* = 3. **(f)** The rates of live and dead cells (%) are based on cell count data on day 7 with medium exchange after culturing 15M66 cells on CDSD-500, CDSD-1000, CDSD-1500, and CDSD-2000 plates. One plate was analyzed for each culture condition. *n* = 3. **(g)** As a control, the numbers of live and dead cells based on cell count data on day 4 with medium exchange after culturing iPSCs (15M66) seeded at 5 × 10^4^ cells/well on an iMatrix-511-coated plate are shown. One plate was analyzed. *n* = 3.

We seeded 15M66 cells onto dot pattern plates (5 × 10^4^ cells per well/six-well plate) in 12 mL/well StemFitAK03 medium containing 10 μM Y-27632. The cells were cultured for 7 days without changing the medium. Cell numbers were counted on day 7, and an mRNA analysis was performed (Fig. 2a, b). Control cells were seeded onto an iMatrix-511-coated plate and cultured for 7 days with standard medium exchange. The data on cell numbers were *t*-tested; comparisons showing significant differences are indicated in Figure 2b. The total number of cells on day 7 was significantly higher on CDSD-500 and CDSD-1000 plates than the CDSD-1500 plate. CDSD-500 plates have small dots and about 10 times as many dots (*n* = 638) than CDSD-1500 plates (*n* = 69). We speculate that the proportion of cells that were able to immediately attach to the dots after seeding was higher for the CDSD-500 plates. CDSD-1500 and CDSD-2000 plates had significantly fewer dead cells on day 7 of culture than the CDSD-500 plate (Fig. 2a). Expression of *OCT3/4* and *NANOG* was higher on all dot pattern plates than in the culture using iMatrix-511 (Fig. 2b).

### Cell numbers and expression of undifferentiated markers in 7-day cultures with medium exchange

We immunostained the cells after 7 days of culture (Fig. 2c). We measured the areas of undifferentiated cells identified by KP-1 staining. The cells cultured on the CDSD-2000 plate had a significantly higher fluorescence staining luminescence area for KP-1-positive cells than the CDSD-500 plate (Fig. 2d). The cells were also subjected to an mRNA expression analysis to compare marker mRNA expression levels of cell growth factors with undifferentiated marker mRNA expression levels (Fig. 2e). The proportions of live and dead cells are shown in Figure 2f. 15M66 cells on day 4 after seeding (5 × 10^4^ cells/well) on iMatrix-511-coated plates were used as controls (Fig. 2g). For densely seeded cells (5 × 10^4^ cells/well), the rate of dead cells increased in the culture group using iMatrix-511 on day 7 of culture. Cell viability was ≥85% in cultures using dot pattern plates (Fig. 2f, g). The 2000 μm giant colony was highly undifferentiated, with low rates of apoptosis and dead cells on dot pattern plates. In the 2000 μm giant colony with high cell proliferation, the levels of *OCT3/4* and *NANOG* mRNA were higher.

### Culture with medium volume kept constant or reduced to half or a quarter

15M66 cells (5 × 10^4^ cells/well) were seeded onto a dot pattern plate in StemFit AK03 (1.5 mL/well) plus 10 μM Y-27632. Every 24 h after cell seeding, the medium in the well was aspirated, and 2.0 mL of StemFitAK03 was added to the well at a 1/1 volume, 1.0 mL at a 1/2 volume, and 0.5 mL at a 1/4 volume.

**FIG. 3.**
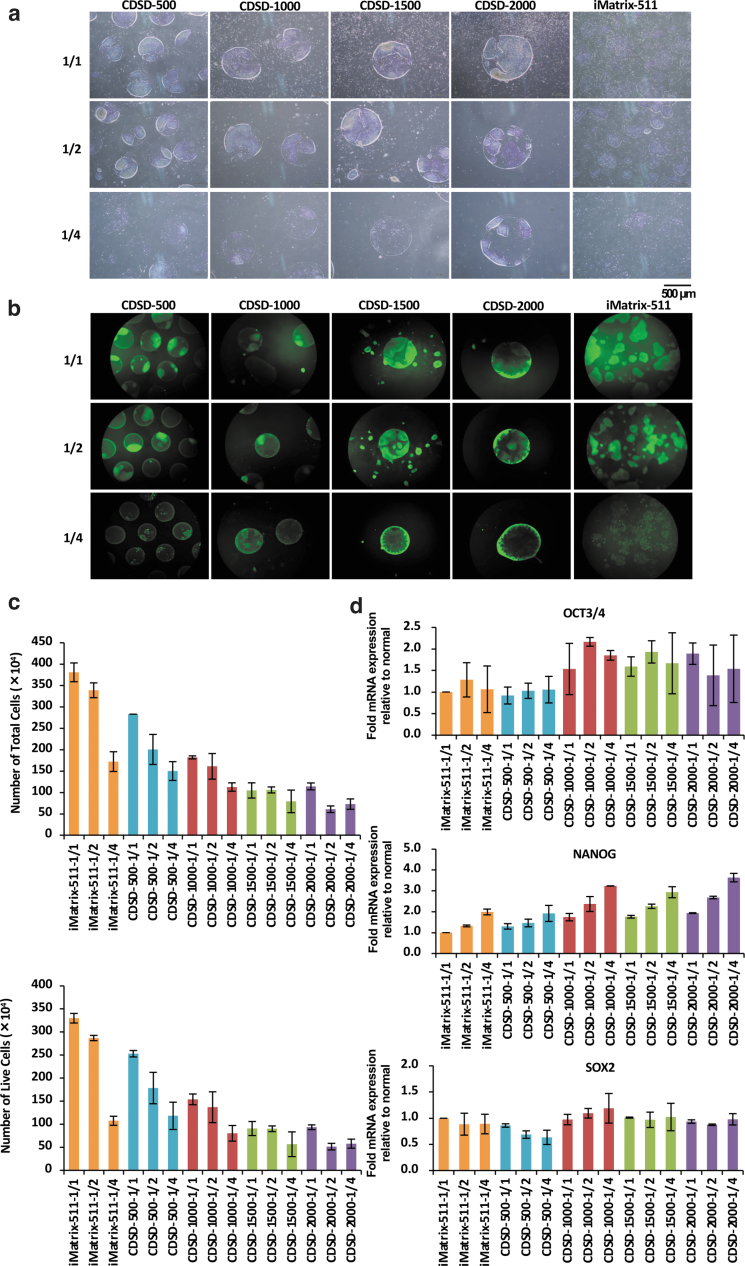
Cultures with constant (1:1) exchange of fresh medium or reduced (1/2, 1/4) volume of medium exchange. **(a)** Light microscope images of iPSCs (15M66) cultured on CDSD-500, CDSD-1000, CDSD-1500, CDSD-2000, or iMatrix-511-coated plates for 5 days with an equal volume of medium exchange (*upper panel*), or with 1/2 the volume exchanged (*middle panel*), or 1/4 the volume exchanged (*lower panel*). Scale bar = 500 μm. **(b)** iPSCs (15M66) in cultures with a constant volume of medium (*upper panel*), 1/2 volume (*middle panel*), and 1/4 volume (*lower panel*) on CDSD-500, CDSD-1000, CDSD-1500, CDSD-2000, and iMatrix-511-coated plates for 5 days. Fluorescence microscope images are shown. Scale bar = 500 μm. **(c)** iPSCs (15M66) were seeded on CDSD-500, CDSD-1000, CDSD-1500, CDSD-2000, and iMatrix-511-coated plates (5 × 10^4^ cells/well) in StemFit AK03 (1.5 mL/well) with 10 μM Y-27632. From the next day, the medium was changed daily with 2.0 mL (constant culture volume), 1.0 mL (1/2 volume), or 0.5 mL (1/4 volume) mL StemFit AK03. The total number of cells (*upper panel*) and the number of live cells (*lower panel*) on day 5 are shown. Two plates were established for each treatment condition. *n* = 2. **(d)** Cultures were established as described in (c) and subjected to real-time PCR of *OCT3/4* (*upper panel*), *NANOG* (*middle panel*), and *SOX2* (*lower panel*). *n* = 2.

Optical micrographs and fluorescence-stained images of KP-1-positive cells on day 5 after cell seeding are shown (Fig. 3a, b). The total number of cells and the numbers of live and dead cells were counted on day 5 (Fig. 3c). In cultures using iMatrix-511 and dot pattern plates, the total number of cells and number of viable cells decreased when the medium volume was reduced by half and further decreased at one quarter medium compared with the original full volume. The total number of cells and number of viable cells were lower in all pattern plates compared with the culture using iMatrix-511; this may have been due to the low total adhesive surface (1.2 cm^2^) of the dot pattern plates. Among the dot pattern plates, the total number of cells and number of viable cells were highest on CDSD-500 plates and lowest on CDSD-2000 plates. On the CDSD-1000 and CDSD-1500 plates, the viable cell count was maintained at 1 mL/day of medium (Fig. 3c).

The results of an mRNA analysis of *OCT3/4*, *NANOG*, and *SOX2* on day 5 are shown in Figure 3d. In the analysis of cells surviving starvation conditions, the half or quarter volume of medium, the levels of *OCT3/4* and *NANOG* mRNA were higher on CDSD-1000, CDSD-1500, and CDSD-2000 plates than in the culture group using iMatrix-511 (Fig. 3d).

Regarding why OCT3/4 and *NANOG* mRNA levels were higher in CDSD-1000 than in CDSD-500 and in CDSD-1500 than in CDSD-2000 dot pattern plates under starvation conditions, first, with normal culture protocols, the *OCT3/4* mRNA expression was higher with larger dot pattern sizes at days 4, 6, and 8 after cell seeding (Fig. 1e). In contrast, with those same culture protocols, the *NANOG* mRNA expression was higher at day 4 after cell seeding when the dot pattern size was smaller (Fig. 1e, left panel) and higher at day 8 after cell seeding when the size of the dot pattern was larger (Fig. 1e, right panel). This result indicates that the mRNA expression of OCT3/4 and NANOG decreases when the colonies stop expanding when the dot pattern size is small, a finding similar to those for iPSCs that reached confluence 8 days after cell seeding (Fig. 1e, right panel).

In the next experiment, in which the medium was not changed for 7 days, the total and viable cell counts were lower or significantly lower in CDSD-1500 and CDSD-2000, where the dot size was ≥1500 μm, compared to CDSD-500 and CDSD-1000, where the dot size was ≤1000 μm; these results differed from those of experiments in which the medium was changed over the next 7 days. This means that the expression of cell growth factors (Cyclin D1, Ki67) was higher in CDSD-2000 than in CDSD-500 when the medium was changed (Fig. 2e), and the expression of Kyoto Probe 1, a marker for undifferentiated cells, was also higher in CDSD-2000 than in CDSD-500 (Fig. 2d). In contrast, the mRNA expression of OCT3/4 and NANOG was significantly lower in CDSD-2000 than in CDSD-1500 in experiments where the medium was not changed for 7 days, indicating that CDSD-2000 results in a lower expression of undifferentiated markers in iPSCs than CDSD-1500 when the medium is not changed for 7 days.

The expression of undifferentiated markers was higher in CDSD-500 and CDSD-1000 and CDSD-1500 with fewer viable cells (Fig. 2a, middle panel) (Fig. 2b, left and middle panels). In CDSD-2000, the number of viable cells was higher (Fig. 2a, middle panel) and the expression of undifferentiated markers lower (Fig. 2b, left and middle panels) than in CDSD-1500.

In conclusion, under starvation experimental conditions, iPSCs on CDSD-2000 were more prone to lower degrees of undifferentiation due to the partial presence of cells on nondots that failed to adhere to the dots during growth of multiple iPSCs adhering to the large dots. In the absence of medium exchange, the medium concentration of proteins essential for maintaining undifferentiation, for example, FGF2, is likely to be reduced, and the undifferentiated level of iPSCs may be unable to be maintained, leading to a decrease in the degree of undifferentiation.

### Induction of cell differentiation to evaluate the quality of the undifferentiated iPSCs on dot pattern plates

The present study investigated the ability of iPSCs on the dot pattern to induce differentiation of the three germ layers of iPSCs—mesoderm cardiomyocytes (Fig. 3a, b), endoderm hepatoblasts (Fig. 3c, d), and ectoderm neural progenitors (Fig. 3e, f)—for 11 days. iPSCs on iMatrix-511 were used as a control for differentiation induction. To confirm that cell differentiation had been induced normally, iPSCs on iMatrix-511 and iPSCs on plates fully coated with CDS were sampled on day 4 after the start of differentiation induction and used as a control for the differentiation induction evaluation. At 7 days after seeding 15M66 cells (5 × 10^4^ cells/well), onto a dot pattern plate, the cells were induced to differentiate into different cell lineages.

**FIG. 4.**
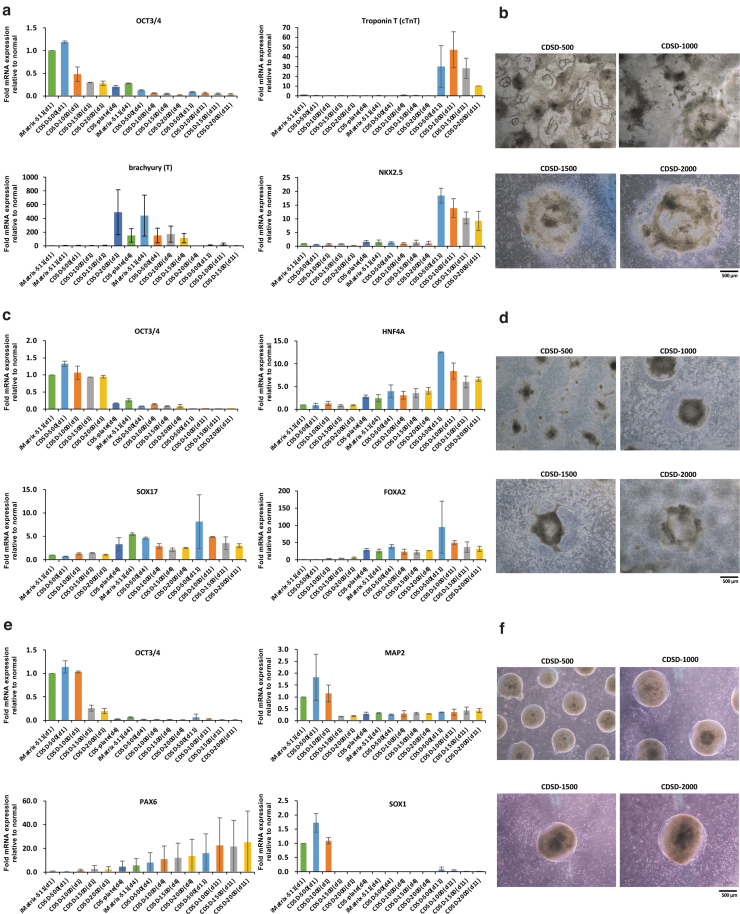
Induced differentiation into the three germ layers. **(a)** iPSCs (15M66) were seeded on CDSD-500, CDSD-1000, CDSD-1500, and CDSD-2000 plates (5 × 10^4^ cells/well), and differentiation into cardiomyocytes was induced from day 7. mRNA analysis data on days 1, 4, and 11 after the induction of cardiac differentiation are shown. The expression of *OCT3/4* (*upper left panel*), Troponin T (*upper right panel*), brachyury (*lower left panel*), and *NKX2.5* (*lower right panel*) is shown. Two plates were established for each condition. As control, a plate (CDS plate) in which the surface treatment of the *dots* was performed on the entire *bottom* surface of the well was used. A control culture using iMatrix-511 (4.6 μL of medium added) was also set up. *n* = 2. **(b)** Images taken with an optical microscope on Day 11 after the induction of differentiation are shown. Scale bar = 500 μm. **(c)** iPSCs (15M66) were seeded on CDSD-500, CDSD-1000, CDSD-1500, and CDSD-2000 plates (5 × 10^4^ cells/well), and differentiation into hepatocytes was induced from day 7. An mRNA analysis was performed on days 1, 4, and 11 after the induction of hepatocyte differentiation. The expression of *OCT3/4* (*upper left panel*), *HNF4A* (*upper right panel*), *SOX17* (*lower left panel*), and *FOXA2* (*lower right panel*) is shown. Two plates were established for each condition. As control, a plate (CDS plate) in which the surface treatment of the *dots* was performed on the entire *bottom* surface of the well was used. A control was set up using cells cultured on an iMatrix-511-coated plate (4.6 μL of medium added). *n* = 2. **(d)** Images taken with an optical microscope on day 11 after the induction of differentiation are shown. Scale bar = 500 μm. **(e)** iPSCs (15M66) were seeded on CDSD-500, CDSD-1000, CDSD-1500, and CDSD-2000 plates (5 × 10^4^ cells/well), and differentiation into neurons was induced from day 7. An mRNA analysis was performed on days 1, 4, and 11 after the induction of neuronal differentiation. The expression of *OCT3/4* (*upper left panel*), *MAP2* (*upper right panel*), *PAX6* (*lower left panel*), and *SOX1* (*lower right panel*) is shown. Two plates were established for each condition. As control, a plate (CDS plate) in which surface treatment of the *dots* was performed on the entire *bottom* surface of the well was used. A control was established by culturing cells on iMatrix-511-coated plates (4.6 μL of medium added). *n* = 2. **(f)** Images taken with an optical microscope on Day 11 after the induction of differentiation are shown. Scale bar = 500 μm.

First, we induced differentiation of cardiomyocytes. mRNA analyses were performed on days 1, 4, and 11 of differentiation (Fig. 4a). As control, a plate for which surface treatment of the dots was performed for the entire bottom surface of the well (CDS plate) was used. As a control, differentiation induction was also performed using cells cultured on iMatrix-511. Optical microscope images ( × 40) of cells on day 11 of induction are shown in Figure 4b. Cells cultured on CDSD-1000 plates showed the highest expression of the myocardial maturation marker *cTnT* on day 11 and had a high degree of differentiation maturity (Fig. 4a, upper right panel). On Day 11, cells on the CDSD-500 plate had the highest expression of the myocardial precursor marker *NKX2*.5 and showed delayed differentiation induction (Fig. 4a, lower right panel). The results of real-time qPCR for mesoderm are shown (*n* = 2). The mesoderm marker T (Brachyury) was increased 8.39-fold in cells cultured on the CDSD-1500 plate on day 11 of differentiation induction compared to controls. Alpha cardiac muscle 1 (ACTC1) was increased 3.02-fold in cells cultured on the CDSD-500 plate, increased 24.41-fold in cells cultured on the CDSD-1000 plate, increased 194.51-fold in cells cultured on the CDSD-1500 plate, and increased 64.47-fold in cells cultured on the CDSD-2000 plate on day 11 of differentiation induction compared to controls.

The DES gene provides instructions for making a protein called desmin, found in heart (cardiac) muscle. DES was increased 8.34-fold in cells cultured on the CDSD-1000 plate and increased 4.21-fold in cells cultured on the CDSD-1500 plate on day 11 of differentiation induction compared to controls. Natriuretic peptide precursor-A (NPPA) is an early and specific marker for the functional myocardium of the embryonic heart. NPPA was increased 9.82-fold in cells cultured on the CDSD-1500 plate on day 11 of differentiation induction compared to controls (Supplementary Fig. S2a).

Next, we sought to induce differentiation into definitive endoderm. An mRNA analysis was performed on days 1, 4, and 7 of differentiation (Fig. 4c). As control, a plate for which surface treatment of the dots was performed for the entire bottom surface of the well (CDS plate) was used. As a control, differentiation induction was also performed using cells cultured on iMatrix-511. Optical microscope images ( × 40) of cells at day 7 are shown in Figure 4d. Cells on CDSD-500 and CDSD-1000 plates had high expression of the endoderm markers *SOX17* (Fig. 4c, lower left panel) and *FOXA2* (Fig. 4c, lower right panel) and of the hepatocyte marker *HNF4A* (Fig. 4c, upper right panel). The results of real-time qPCR for mesoderm are shown (*n* = 2). The endoderm marker Forkhead box protein A2 (FOXA2) was increased 64.05-fold in cells cultured on the CDSD-500 plate, increased 64.35-fold in cells cultured on the CDSD-1000 plate, increased 31.74-fold in cells cultured on the CDSD-1500 plate, and increased 47.89-fold in cells cultured on the CDSD-2000 plate on day 11 of differentiation induction compared to controls.

The endoderm marker GATA binding protein 4 (GATA4) was increased 16.00-fold in cells cultured on the CDSD-500 plate, increased 7.91-fold in cells cultured on the CDSD-1000 plate, increased 7.95-fold in cells cultured on the CDSD-1500 plate, and increased 12.02-fold in cells cultured on the CDSD-2000 plate on day 11 of differentiation induction compared to controls, and GATA binding protein 6 (GATA6), a marker of the endodermis, was increased 4.03-fold in cells cultured on the CDSD-500 plate on day 11 of differentiation induction compared to controls (Supplementary Fig. S2b).

Finally, we sought to induce differentiation into neuroprogenitor cells. As a control, differentiation induction was also performed using cells cultured on iMatrix-511. An mRNA analysis was performed on days 1, 4, and 7 (Fig. 4e). Optical microscope images ( × 40) of cells at day 7 are shown in Figure 4f. Cells cultured on the CDSD-2000 plate showed higher expression of the neural stem cell marker *PAX6* than those on the CDSD-500 plate (Fig. 4e, lower left panel).

The results of real-time qPCR for mesoderm are shown (*n* = 2). The ectoderm marker paired box 6 (PAX6) was increased 4.54-fold in cells cultured on the CDSD-1000 plate, increased 6.05-fold in cells cultured on the CDSD-1500 plate, and increased 6.00-fold in cells cultured on the CDSD-2000 plate on day 11 of differentiation induction compared to controls. Nestin is a protein encoded by the NES gene in humans. These intermediate filament proteins are expressed mostly in nerve cells, where they are implicated in the radial growth of the axon. NES was increased 3.02-fold in cells cultured on the CDSD-1500 plate and increased 2.00-fold in cells cultured on the CDSD-2000 plate on day 11 of differentiation induction compared to controls (Supplementary Fig. S2c).

## Discussion

We belong to the Research and Development Department, which carries out basic and applied research, accumulates data, and then conducts bridging research for clinical applications. Even in joint research with companies, such as in this article, we start with experiments in the Research and Development Department. In contrast, the department that manufactures clinical iPSCs is the manufacturing department, similar to a factory in the industrial field. The first step in proposing a new production method for clinical iPSCs is for the Research and Development Department to conduct basic and applied research, which then contributes to the publication of academic articles, as this article has done. Bridging research is then performed, in which the manufacturing department follows the experimental protocols of the academic article using clinical iPSC lines. As the number of generations of clinical iPSC lines is controlled, one vial of clinical iPSC lines must be shipped and used in the manufacturing department when conducting bridging research. When a new manufacturing method for clinical iPSCs is proposed, the name of the cell line and the number of passages must be specified, so data obtained by culturing clinical iPSC lines for more than the specified number of passages cannot be used by the manufacturing department.

We previously reported on the protocol for the generation of clinical iPSC lines.^3,13^ In the present experiment, we used the iPSC line 15M66, which was established using the same protocol as the clinical iPSC line and is owned by the Research and Development Department. We only used the iPSC line 15M66 in the present study to transfer the protocol to the production department for publication in this article. However, the protocol described in this article is specific to our previously reported bridging study on the process of manufacturing iPSC lines for clinical use and is a technology that has been evaluated for a limited number of iPSC lines.

Both research and clinical iPSCs are pluripotent, so naturally, if undifferentiated iPSCs are injected into mice, a teratoma will form. Therefore, undifferentiated iPSCs cannot be used as therapeutic cells in an untreated state. Undifferentiated iPSCs must first be treated for differentiation induction and fully induced into differentiated cells. A number of methods with high differentiation induction efficiency have already been reported as protocols for inducing the differentiation of iPSCs for clinical use.^10,14,15^ Even if the differentiation induction efficiency is not perfect, methods to increase the purity of differentiated cells using a cell sorter have also been reported.^16^ We also previously reported a method for separating undifferentiated iPSCs from differentiated cells that have been induced to differentiate.^17^

We also attempted incubation periods longer than 8 days to further the value of this technology. However, incubations beyond 8 days resulted in colonies that were larger than the size of the pattern. Therefore, iPSCs need to be single cells seeded at the center of the dot pattern. We did consider seeding iPSCs at arbitrary locations using a single-cell seeding device, but the introduction of a single-cell seeding device into a cell culture processing facility and strict control of seeding points require extremely sophisticated technology. Therefore, in this article, the culture period was limited to 8 days. As a result, the iPSC seeding points on the dot pattern are the sites where cells spread at the seeded cell density and spontaneously adhered.

The protocols that enable iPSCs to be cultured for long periods have a marked influence on cell production and the use of the cells. For example, if the interval between cell passages can be extended, then opportunities for carrying out cell treatments at each passage are also extended. In this study, we investigated the effects of limitation of the cell adhesion area using culture plates in which the cell adhesion was restricted to a 1.2-cm^2^ area in the 9.6 cm^2^ bottom of a six-well plate. Thus, iPSCs seeded onto the plate were able to adhere and grow only in 12.5% of the total area of the well bottom. Finally, it was possible to achieve an eight-fold increase in the amount of medium relative to the area of cell adhesion. For iPSCs in colonies of diameter 500–2000 μm and a cell adhesion area of 1.2 cm^2^, 1 × 10^6^ cells were obtained 6–8 days after cell seeding (Fig. 1d). Analysis of mRNA expression in colonies of diameter 500–2000 μm showed that *OCT3/4* expression was higher than in iPSCs cultured on control iMatrix-511-coated plates, even at 8 days after seeding (Fig. 1e).

Two factors influence the differences between the control and dot pattern plates: (1) the different characteristics of iMatrix-511 and fibronectin motif scaffolds; and (2) the size of the dot pattern. In a previous study, we examined the first factor by comparing cultures using iMatrix-511 plates and StemFit medium and cultures using vitronectin and E8 medium.^8^ In this study, therefore, we focus on the second factor, namely the size of the culture areas.

Previous reports have shown that iPSCs passaged at 4-day intervals on a 1 mm circular dot-coated vitronectin scaffold produced stable colonies.^18^ On this basis, a protocol was developed in which cell culture was initiated at a high cell seeding concentration (3–4 × 10^5^ cells/well), and passage was performed at 4 days after seeding.^18^ Passaging cells at 4-day intervals appears to contribute to maintaining the condition of iPSCs by preventing cell death. This report is consistent with our findings here.

The development of the appropriate technology for supporting stable long-term culture of iPSCs is required for the industrial production of iPSCs. However, conventional culture methods do not allow iPSCs to be passaged for more than a week. This is thought to be because when iPSC colonies grow and adhere to surrounding colonies, the cells become overcrowded, causing cell death (Fig. 1d). In the present study, we demonstrate that iPSCs cultured on dot pattern plates can tolerate culture for up to 8 days of passaging (Fig. 1d, and e) and also tolerate culture under starvation conditions (where the medium supply is low) (Fig. 2a, b). The undifferentiated iPSCs in these cultures were still alive. We previously showed that the signaling pathways that sustain iPSC survival are particularly strongly influenced by the PI3K/AKT signaling pathways and that PIK3CA plays a major role in this pathway.^17^ However, the expression of *PIK3CA* does not appear to be significantly different between cultures grown on iMatrix-511 and those on dot pattern plates (Supplementary Fig. S1c).

We compared the characteristics of iPSCs on iMatrix-511-coated dot pattern plates using mRNA arrays for gene markers of pluripotency, the cell cycle, and fibroblast growth factor (Supplementary Fig. S1a–c). We found that markers for the undifferentiated state, *OCT4* (*POU5F1*), *NANOG*, and *DNMT3B*, were all highly expressed in cells on a CDSD-2000 plate (Supplementary Fig. S1a), a finding consistent with the data presented earlier (Fig. 2d, e). In addition, the expression of *NEUROD*, which is related to the development of the nervous system and pancreas, was high in cells on the CDSD-2000 plate (Supplementary Fig. S1a); this is consistent with the fact that the CDSD-2000 plate had a differentiation-inducing effect on ectoderm neural progenitor cells (Fig. 4e).

In contrast, *AFP* and *HNF4A*, which are markers of hepatic progenitor cells, were highly expressed in cells on the CDSD-2000 plate (Supplementary Fig. S1a). However, after inducing differentiation of hepatic progenitor cells, expression of *HNF4A* was higher in cells on the CDSD-500 plate than on the CDSD-2000 plate. In addition, *T* and *NKX2*.5, which are myocardial markers expressed by iPSCs, were highly expressed on CDSD-2000 (Supplementary Fig. S1a). Expression of *T* and *NKX2*.5 after induction of myocardial differentiation was lower in cells cultured on the CDSD-2000 plate than on the CDSD-500 plate (Fig. 4a).

These results indicate that the differentiation markers expressed by undifferentiated iPSCs cannot be used as an accurate indicator of the ability to induce differentiation to ectoderm/mesoderm/endoderm. The phenomenon in which iPSCs spontaneously differentiate without being induced to do so is termed the epithelial–mesenchymal transition.^19^ The expression of *SNAIL* and *VIMENTIN* is seen when the area around the iPSC colony begins to differentiate.^20,21^ Our mRNA array results show that iPSCs cultured on dot pattern plates have a lower *VIMENTIN* expression than those on a iMatrix-511 plate (Supplementary Fig. S1a, and c). This result was confirmed through staining with KP-1: iPSCs cultured on dot pattern plates showed a higher level of staining than those on the iMatrix-511 plate, even under starvation conditions (Fig. 3b). This result demonstrates that iPSCs cultured on dot pattern plates retained the ability to maintain the undifferentiated state.

Compared to protocols using iMatrix-511 plates, which are currently used for culturing iPSCs in the clinical setting, cultures using dot pattern plates can produce undifferentiated cells with a high survival rate for over 1 week after passage. It was possible to maintain cells in culture under these conditions. Our mRNA array analysis indicated that the characteristics of iPSCs cultured on dot pattern plates were similar to those on iMatrix-511 plates.

If iPSCs can be cultured for more than 1 week after passaging, the need to perform Saturday and Sunday cell culture processing operations in the production department [cell production plant] could be eliminated. However, when using this technology with a limited culture area, the cell yield is constant, regardless of the cell growth rate. Therefore, the cost of culturing iPSCs per unit day is considered to increase in proportion to the restricted culture area compared to 2D or 3D culture, where the culture area is not restricted. Nevertheless, we consider this technology, which can suppress labor costs, the largest expenditure in iPSC production, to have technical advantages that will contribute to the further development of the cell processing industry using iPSCs. As a technology that can culture iPSC colonies at regular intervals, this technology can contribute to the stabilization of industrial iPSC culture methods as a method of process control.

In conclusion, dot pattern plates appear to be suitable for long-term culture of cells, making it possible to flexibly adjust the manufacturing process of iPSCs. The scaffold is made from a polymer that shows high biocompatibility and solubility in organic solvents. This polymer scaffold has the potential to coat many shapes of cultureware with the use of a general coating method. In contrast, conventional protein scaffolds such as iMatrix-511 (laminin), vitronectin, and fibronectin are water soluble and are therefore used as plate-coating agents in aqueous solution. We were unable to form dot patterns on the plates with water-soluble proteins in this study. In conclusion, this study demonstrated the geometric dot size effect of the iPSC culture process. Dot pattern plates appear to be suitable for the long-term culture of cells, thus making it possible to flexibly adjust the iPSC manufacturing process.

## Supplementary Material

Supplemental data

Supplemental data
