# Supplementary material for: Improved Production of Induced Pluripotent Stem Cells Using Dot Pattern Culture Plates
Source: Tissue Eng Part C Methods. 2023 Sep 14;29(9):410–23. doi: 10.1089/ten.tec.2023.0068 (PMC10517333; doi:10.1089/ten.tec.2023.0068)
Supplement: Supplemental data [file Suppl_FigureS2.docx]

**Supplementary Figure 2. The analysis of differentiated induced cells using a qPCR array.** iPSCs (15M66) were seeded on CDSD-500, CDSD-1000, CDSD-1500, and CDSD-2000 plates (5×10^4^ cells/well), and differentiation into cardiomyocytes was induced from day 7. mRNA analysis data on day 11 after the induction of cardiac differentiation are shown. Control culture using iMatrix-511 (4.6 μl of medium added) was also established. mRNA was extracted, and cDNA was synthesized from the cells. The results of the mRNA expression analysis are shown. The expression was calculated using the ΔΔCt method. The target gene expression was corrected based on housekeeping gene expression. Data were normalized by converting the average expression of various mRNAs in the control to 1. Results of the real-time qPCR analysis for mesoderm (a), endoderm (b) and ectoderm (c) are shown (n = 2).
