# Supplementary material for: Improved Production of Induced Pluripotent Stem Cells Using Dot Pattern Culture Plates
Source: Tissue Eng Part C Methods. 2023 Sep 14;29(9):410–23. doi: 10.1089/ten.tec.2023.0068 (PMC10517333; doi:10.1089/ten.tec.2023.0068)
Supplement: Supplemental data [file Suppl_FigureS1.docx]

**Supplementary Figure 1. qPCR array analysis of iPSCs (15M66) cultured on pattern plates.** cDNAs were synthesized from iPSCs (15M66) cultured on CDSD-500 and CDSD-1000 plates for 7 days with medium exchange after cell seeding (5×10^4^ cells/well). As a control, cells (5×10^4^ cells/well) were seeded onto an iMatrix-511-coated plate; the numbers of live and dead cells are shown on day 4. Gene expression level was calculated using the ΔΔCt method. The expression of the target gene was corrected against the expression of the housekeeping gene. (a) Quantitative real-time PCR of pluripotency. One plate was established for each condition. n = 1. (b) Quantitative real-time PCR of the cell cycle. One plate was established for each condition. n = 1. (c) Quantitative real-time PCR of fibroblast growth factor. One plate was established for each condition. n = 1.
